# Non-invasive Liver Fibrosis Scores Are Associated With Recurrence of Postoperative Chronic Subdural Hematoma

**DOI:** 10.3389/fneur.2022.873124

**Published:** 2022-06-13

**Authors:** Peng Zhang, Hua Wang, Han Bao, Ning Wang, Zhen Chen, Qi Tu, Xiao Lin, Yun Li, Zezheng Zheng, Yu Chen, Linhui Ruan, Qichuan Zhuge

**Affiliations:** ^1^Department of Neurosurgery, The First Affiliated Hospital of Wenzhou Medical University, Wenzhou, China; ^2^Zhejiang Provincial Key Laboratory of Aging and Neurological Disorder Research, The First Affiliated Hospital of Wenzhou Medical University, Wenzhou, China; ^3^Neurointerventional Department, Zhejiang Hospital, Hangzhou, China

**Keywords:** chronic subdural hematoma, liver fibrosis score, recurrence, risk factors, aspartate aminotransferase/alanine aminotransferase ratio, fibrosis-4, forns index

## Abstract

**Objective:**

Although liver diseases have already been identified as a risk factor for increased recurrence and mortality in patients with chronic subdural hematoma (CSDH), the association between subclinical liver disease, specifically liver fibrosis (LF), and CSDH remains unknown. In the present study, we aimed to investigate the association between the LF scores and CSDH recurrence.

**Methods:**

We retrospectively analyzed consecutive patients with CSDH who underwent burr-hole irrigation in the First Affiliated Hospital of Wenzhou Medical University between January 2015 and December 2018. The clinical data were collected, and the LF scores were calculated including aspartate aminotransferase–platelet ratio index (APRI), fibrosis-4 (FIB-4), and Forns index. Multivariable logistic regression analysis was applied to identify the association between the LF scores and CSDH recurrence, and Cox regression model and Fine–Gray competing risks model were performed to calculate hazard ratios (HRs) for CSDH recurrence based on time-to-event outcomes. The C-statistic, the integrated discrimination improvement (IDI), and the net reclassification improvement (NRI) evaluated the additive value of the LF scores to predict the recurrence of CSDH.

**Results:**

A total of 419 patients with CSDH were included, hematoma recurrence was observed in 62 patients (14.80%) within 1 year after surgery. The LF scores were significantly higher in those who recurred, whereas the standard hepatic assays were mostly normal. The patients were assigned to groups of high and low LF scores based on the validated cut-offs; compared with the subjects with low scores, those with high score levels had significantly higher recurrence rates. After adjusting for potential confounders, the LF scores were independently associated with CSDH recurrence, multivariable-adjusted HRs (95% CI) for those with higher levels of APRI, FIB-4, and Forns score were 4.32 (1.37–13.60), 2.56 (1.20–5.43), and 2.02 (1.07–3.79) for the recurrence of CSDH, respectively. Moreover, adding the APRI to the conventional model improved the C-statistic from 0.731 to 0.763, with an NRI and IDI of 7.50 and 1.35%, respectively. Two further commonly-used LF score indices (FIB-4 score and Forns index) yielded comparable results.

**Conclusions:**

The data from this study first indicated that the high LF scores were significantly associated with the recurrence of CSDH and that careful follow-up in these patients may be needed.

## Introduction

Chronic subdural hematoma (CSDH) is a common neurologic disorder that occurs in 1–13.1 per 100,000 persons per year (1). Its morbidity rate is significantly increasing because of the aging population and is expected to double in the future decades (2, 3). The current treatment for CSDH follows various approaches whereas surgical evacuation of the subdural collection remains the main treatment approach for the symptomatic patients (4). Burr-hole irrigation (BHI) is the preferred technique for treating CSDH with a relatively good outcome; however, the hematoma recurs in 10–20% of surgically treated patients (5–7). In the current literature, a complex intertwining pathway of inflammation, angiogenesis, local coagulopathy, recurrent microbleeds, and exudates has been suggested to be associated with the progression and recurrence of CSDH (3, 8).

Liver is known for diverse functions, some of which may be related to hemorrhage (9, 10). The previous studies demonstrated that liver disease is significantly associated with the recurrence of CSDH, and patients with coagulopathy and liver disease are at greater risk for recurrence than those with coagulopathy alone (11, 12). Furthermore, Chen et al. (13) reported liver disease—particularly cirrhotic liver disease—was a serious comorbidity with a poor prognosis in patients with CSDH, markedly increased the surgical mortality and postoperative recurrence of CSDH. Despite these previous data highlight a possible link between advanced liver disease and CSDH recurrence, it is unclear if these findings also apply to subclinical liver disease. Liver fibrosis (LF)—an often clinically silent manifestation of chronic liver disease and a histological precursor to cirrhosis—is present in up to 9% of individuals without known liver disease (14–16). Emerging evidence suggests that LF is the key predictor of adverse prognosis in patients with coronary artery disease (CAD) (17), and also significantly associated with postinterventional hemorrhagic transformation in acute ischemic stroke and substantial hematoma expansion in primary intracerebral hemorrhage (18, 19). However, data are lacking regarding the implications of LF for patients with CSDH. Even though the golden standard for diagnosis of LF is liver biopsy, it cannot be performed on all patients for fibrosis screening (17–20). Non-invasive scoring systems calculated using routinely available clinical and laboratory parameters might be a safe and easily assessable alternative for the initial evaluation of fibrosis, especially in subjects without symptoms or in the subjects with history of liver diseases. Moreover, these LF scores have been validated to estimate advanced fibrosis with high sensitivity and specificity, not only in patients with liver disease but also in the general population.

Currently, several practical clinic–laboratorial scores have been reported to predict the LF probability, including aspartate aminotransferase–platelet ratio index (APRI) (17, 19), gamma–glutamyltransferase platelet ratio (GPR) (17), non-alcoholic fatty liver disease fibrosis score (NFS) (19), fibrosis-4 (FIB-4) score, and Forns index score (17, 21). Among all the validated non-invasive markers, APRI, FIB-4, and Forns index scores are the three most efficient indices to identify those with high probability of advanced fibrosis, with area under the receiver operating characteristic (ROC) curve values ranging from 0.76 to 0.88 (19, 22, 23). Furthermore, the recent studies had indicated that non-invasive LF scores (APRI, FIB-4, and Forns index scores) can predict the bleeding events in patients with cerebrovascular disease, such as postinterventional hemorrhagic transformation in acute ischemic stroke (18), and substantial hematoma expansion in primary intracerebral hemorrhage (19, 24). Hence, we focused mainly on the preceding three non-invasive LF scores and investigated their impact on the recurrence of CSDH.

## Methods

### Patient Population

Patients with CSDH who were admitted to the Department of the Neurosurgery of First Affiliated Hospital of Wenzhou Medical University between January 2015 and December 2018 were included. A diagnosis of CSDH was confirmed by head computed tomography (CT) and/or magnetic resonance imaging (MRI). The following exclusive criteria were used: (1) Younger than 18 years old; (2) conservative treatment or initial surgery in other hospitals; (3) severe renal or blood diseases; (4) severe surgical complications or hospital mortality; (5) lost to follow-up or incomplete clinical data. For this specific investigation, the patients with liver cirrhosis or other potential causes of liver diseases (i.e., viral hepatitis, autoimmune hepatitis, hereditary liver disease, secondary causes of fatty liver, and drug-induced liver disease) were excluded (17, 24, 25). The participants with excessive alcohol consumption (>21 drinks/week in men and >14 drinks/week in women) were also excluded (17, 25, 26). The retrospective study was approved by the Ethics Committee in Clinical Research (ECCR) of the authors' hospitals, and the requirement for informed consent was waived.

### Surgical Procedures and Management

According to our previous report, all patients underwent standard BHI surgery with general anesthesia (27). Briefly, a single burr hole was drilled, and irrigation of the hematoma with normal saline was subsequently performed. After the irrigation, a silicone catheter with a closed drainage system was inserted into the hematoma cavity. Postoperatively, the catheters were withdrawn within 72 h in most cases. The head CT was performed within the first 48 h and on day 6 or 7 after the operation. Pre-existing antiplatelet/anticoagulant therapy (AAT) was discontinued upon admission and re-established 1 week after surgery in case of complete resolution of the hematoma.

### Data Collection

The files of the patients with CSDH were reviewed. Then, the demographic and clinical data were collected the following information: Patient's age, sex, smoking, drinking, comorbidities [hypertension, diabetes mellitus (DM), and CAD], history of head trauma (28), AAT, as well as major presenting symptoms. The hematoma location, radiographic density, and preoperative volume were extracted by reviewing the initial CT scan. The hematoma density was classified as a heterogeneous group including a layering and mixed density type and a homogeneous group including high-density, isodensity, and low-density types (29, 30). The volumes of subdural hematoma were accurately calculated using 3D Slicer (Surgical Planning Laboratory, Harvard University, Boston, MA, USA) software. Moreover, the laboratory parameters on admission were obtained including platelet count, prothrombin time–international normalized ratio (PT–INR), activated partial thromboplastin time (APTT), serum liver enzymes [aspartate aminotransferase (AST), alanine aminotransferase (ALT), gamma-glutamyltransferase (GGT)], and total cholesterol levels.

### Liver Fibrosis Scores

For assessment of LF probability, we primarily analyzed the APRI, a well-validated and clinically established LF scores. The APRI was calculated with following equation, with the cut-off value (> 1.0) as for low- and high-risk categories: APRI = AST (IU/L)/AST (the upper limit of normal, ULN) × 100/platelet count (10^9^/L) (19, 22). To broader analyze our data and confirm the potential associations, we chose to investigate two additional LF scores. These were the FIB-4 and Forns index scores. The FIB-4 was calculated as follows: FIB-4 = [age (years) × AST (IU/L)]/[platelet count (10^9^/L) × √ALT(IU/L)], with cut-offs at 3.25 (19, 22, 23). The Forns index was calculated as follows: 7.811 – 3.131 × log[platelet count (10^9^/L)] + 0.781 × log[GGT (IU/L)] + 3.467 × log[age (years)] – 0.014 × total cholesterol (mg/dl), with cut-offs being set at 6.9 (17, 21–23).

### Recurrence and Follow-Up

The primary outcome was hematoma recurrence up to 1 year (12 months) after the original operation. The routine outpatient visits were scheduled for all patients at 1 month, and 3 months after surgery. Thereafter, clinical and imaging follow-up were individualized; if a physical consultation was not possible, we performed a telephone interview. Similar to the previous studies, the recurrence was defined as an increased volume of the subdural collection and brain compression on the same side as the initial operation, with new or progressing clinical symptoms indicating surgical treatment (31, 32). The time to recurrence was measured in days from the operation day onward (33).

### Statistical Analysis

Continuous variables are presented as mean ± standard deviation (SD) or median [interquartile range (IQR)] as appropriate, according to the distribution of the variables. The categorical variables are described as number (percentage). The differences between groups were determined with independent samples *t*-test, Mann–Whitney U test, χ^2^ test, or Fisher's exact test wherever appropriate. To minimize the bias by confounding, we performed the propensity score matching (PSM) (1:2 match, caliper 0.2) to adjust for imbalances of clinically relevant parameters differing between high and low LF scores groups. Analyses for the recurrence of CSDH were conducted in the PSM cohort.

The cumulative hazard for recurrence was evaluated using the Kaplan–Meier method classified based on the LF scores, with censoring of patients who exhibited no recurrence or recurrence-related symptoms on the last follow-up CT scan or visit during the follow-up period. Multivariable-adjusted hazard ratio (HR) and 95% confidence intervals (CI) were calculated using the Cox regression model. To account for competing risks due to mortality, we fitted a proportional subdistribution hazards regression model for CSDH recurrence with death as a competing event. A cumulative incidence of recurrence was evaluated again using the Gray's test for univariate analysis and the Fine–Gray competing risks model for multivariate analysis (33, 34). Odds ratios (OR) and 95% CI for recurrence risk of the LF scores were determined by performing multivariate-adjusted binary logistic regression. In multivariate regression models, traditional risk factors including age, sex, smoking, drinking, comorbidities, history of head trauma, AAT, symptoms, laboratory investigation, and CT scan hematoma characteristics were used as adjustments. These LF scores were analyzed as continuous variable, per SD increment and categorical variable by conventional cut-offs (described above). We also performed the sensitivity analyses excluding the patients with drinking history in view of some degree of imprecision of self-reported alcohol use. To improve clinical interpretation of results and allow for a non-linear association between the LF scores and hematoma recurrence, we *a priori* categorized the LF scores based on quartiles. To evaluate the trends in categorical values across the quartiles of LF scores, we performed the Cochran–Armitage and Jonckheere–Terpstra tests (35). Restricted cubic splines (RCS) with three knots, adjusted for confounders, were used to detect the dose–response relationship between the LF scores and CSDH recurrence. The ROC curve which is equivalent to the C-statistic was constructed to estimate the discriminative power of the LF scores for CSDH recurrence. To investigate whether the predictive accuracy of CSDH recurrence improves after the addition of the LF scores to a conventional model (CM) that included the aforementioned potential risk factors, we compared the same area under the curve (AUC) between CM and CM plus LF scores, according to the method suggested by Hanley and McNeil (36). We used net reclassification improvement (NRI) with a category-free option and integrated discrimination improvement (IDI) calculations to quantify the improvement in actual reclassification and sensitivity resulting from the addition of the LF scores.

All tests were two sided, and a *p* < 0.05 was considered statistically significant. The statistical analyses were performed with SPSS, version 25.0, software (SPSS, Inc.) and R language, version 4.0.5 (Feather Spray).

## Results

### Patients Characteristics

**Figure 1:**
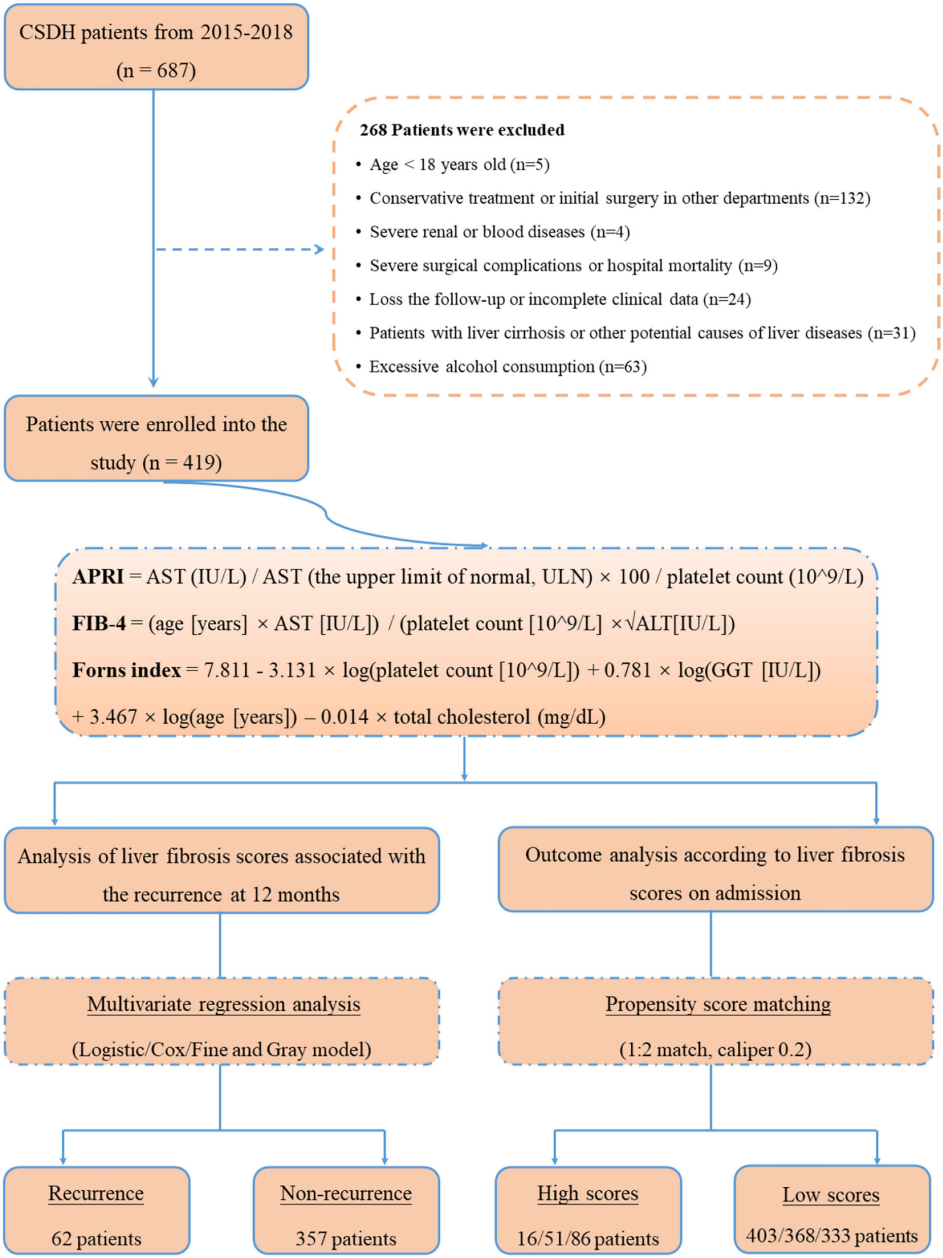
Flowchart of detailed design for the study.

The study flowchart is illustrated in Figure 1. During the study period, a total of 687 consecutive patients with CSDH were assessed initially, and afterwards, 268 patients were excluded in accordance with the exclusion criteria. Ultimately, 419 CSDH cases were enrolled in our study. The median age of participants was 72 years (range 21–91 years), with 15.27% females. All patients were followed up to 1 year (12 months) after surgery for the outcomes of recurrence of CSDH. In total, 10 (2.38 %), 45 (10.73 %), and 62 (14.80 %) patients experienced recurrence of CSDH within 1 month, 3 months, and 12 months after surgery, respectively. The standard liver chemistries and coagulation parameters were generally in the normal range in the study population; 6.21% (*n* = 26) had an AST > 40 IU/L, 4.30% (*n* = 18) had an ALT >40 IU/L, 6.21% (*n* = 26) had a PLT <125 × 10^9^/L, 7.16% (*n* = 30) had a PT–INR >1.15, and 7.88% had an APTT >43 s. The baseline characteristics and outcomes of the patients were presented in Table 1.

**Table 1:** Baseline characteristics and outcomes of 419 patients with CSDH.

**Characteristics**	**Value**
Age (years)	72 (64–80)
**Gender**	
Female	64 (15.27)
Male	355 (84.73)
**Personal/Past history**	
Smoking	168 (40.10)
Drinking	100 (23.87)
Hypertension	169 (40.33)
Diabetes	52 (12.41)
Cardiac diseases	31 (7.40)
Head injury	287 (68.50)
Antiplatelet/anticoagulant therapy	81 (19.33)
**The main symptoms**	
Headache and/or dizziness	235 (56.09)
Limb weakness	190 (45.35)
Disorientation/memory impairment	44 (10.50)
Aphasia	19 (4.53)
Disturbance of consciousness	33 (7.88)
**Unilateral/bilateral hematoma**	
Left	192 (45.82)
Right	142 (33.89)
Bilateral	85 (20.29)
**Hematoma density**	
Homogeneous	292 (69.69)
Heterogeneous	127 (30.31)
Hematoma volume (ml)	108.90 (94.56–124.52)
**Laboratory investigation**	
Total cholesterol (mmol/L)	3.75 (3.24–4.40)
Platelet (×10^9^/L)	214 (174–250)
Platelet (×10^9^/L) <125	26 (6.21)
PT–INR	1.02 (0.96–1.07)
PT–INR >1.15	30 (7.16)
APTT (s)	36.1 (33.4–39.3)
APTT >43 s	33 (7.88)
AST (IU/L)	20 (17–25)
AST >40 IU/L	26 (6.21)
ALT (IU/L)	15 (11–21)
ALT >40 IU/L	18 (4.30)
GGT (IU/L)	14 (10–29)
**Liver fibrosis scores**	
APRI	0.24 (0.18–0.34)
APRI >1	16 (3.82)
FIB-4	1.80 (1.30–2.40)
FIB-4 >3.25	51 (12.17)
Forns index	6.0 (5.0–6.8)
Forns index >6.9	86 (20.53)
**Recurrence at follow-up**	
1 month	10 (2.38)
3 months	45 (10.73)
12 months	62 (14.80)
Death (in 12 months)	11 (2.63)

*Data are expressed as n (%), mean ± standard deviation, or median (interquartile range), as appropriate*.

*CSDH, chronic subdural hematoma; PT–INR, prothrombin time–international normalized ratio; APTT, activated partial thromboplastin time; AST, aspartate aminotransferase; ALT, alanine aminotransferase; GGT, gamma-glutamyltransferase; APRI, indicates Aspartate Aminotransferase–Platelet Ratio Index; FIB-4, Fibrosis-4*.

### Stratification by Liver Fibrosis Scores

The median APRI, FIB-4, and Forns index scores were 0.24 (IQR: 0.18–0.34), 1.80 (IQR: 1.30–2.40), and 6.0 (IQR: 5.0–6.8), respectively. Based on the validated thresholds, there were 16 patients (3.82%) with a high probability of fibrosis by the APRI, 51 patients (12.17%) by the FIB-4, and 86 patients (20.53%) by the Forns index. Supplementary Table S1 outlines the baseline characteristics of those divided according to low and high LF scores. The patients with high LF scores, as expected because of its formula, were older, with greater total cholesterol levels, and had lower platelets, higher serum AST and ALT than those with low LF scores. Moreover, they more frequently had CAD, a history of head trauma and heterogeneous hematoma, with longer PT–INR values. Notably, CSDH recurrence and mortality rates after surgery were more frequent in patients with high LF scores (all *p* < 0.05). To account for confounding bias between patients with low and high LF scores, PSM was performed, after which two evenly balanced cohorts were available for the analyses of hematoma recurrence (Supplementary Figure S1). Regarding the endpoints, we still observed the rate of CSDH recurrence within 12 months of surgery was higher in patients with high LF scores (Table 2).

**Table 2:** Baseline characteristics and outcomes of patients with CSDH, stratified by liver fibrosis scores after propensity score matching.

**Characteristics**	**Low APRI**	**High APRI**	***p***	**Low FIB-4**	**High FIB-4**	***p***	**Low Forns**	**High Forns**	***p***
	***n* = 32**	***n* = 16**		***n* = 102**	***n* = 51**		***n* = 172**	***n* = 86**	
Age	71 (61–76)	76 (73–81)	**0.022**	73 (65–81)	80 (75–85)	**<0.001**	71 (63–79)	77 (71–83)	**<0.001**
**Gender**									
Female	3 (9.38)	2 (12.50)	1.000	19 (18.63)	8 (15.69)	0.653	20 (11.63)	10 (11.63)	1.000
Male	29 (90.62)	14 (87.50)		83 (81.37)	43 (84.31)		152 (88.37)	76 (88.37)	
**Personal/Past history**									
Smoking	14 (43.75)	6 (37.50)	0.679	41 (40.20)	20 (39.22)	0.907	77 (44.77)	40 (46.51)	0.791
Drinking	8 (25.00)	3 (18.75)	0.903	19 (18.63)	8 (15.69)	0.653	58 (33.72)	27 (31.40)	0.708
Hypertension	11 (34.38)	7 (43.75)	0.527	57 (55.88)	26 (50.98)	0.566	72 (41.86)	36 (41.86)	1.000
Diabetes	1 (3.12)	2 (12.50)	0.527	13 (12.75)	8 (15.69)	0.618	24 (13.95)	14 (16.28)	0.619
Cardiac diseases	1 (3.12)	1 (6.25)	1.000	12 (11.76)	9 (17.65)	0.319	17 (9.88)	14 (16.28)	0.136
Head injury	24 (75.00)	11 (68.75)	0.909	60 (58.82)	28 (54.90)	0.644	103 (59.88)	52 (60.47)	0.928
Antiplatelet/anticoagulant therapy	8 (25.00)	4 (25.00)	1.000	26 (25.49)	14 (27.45)	0.795	35 (20.35)	18 (20.93)	0.913
**The main symptoms**									
Headache and/or dizziness	23 (71.88)	10 (62.50)	0.509	58 (56.86)	28 (54.90)	0.818	105 (61.05)	53 (61.63)	0.928
Limb weakness	16 (50.00)	9 (56.25)	0.683	40 (39.22)	21 (41.18)	0.815	67 (38.95)	34 (39.53)	0.928
Disorientation/memory impairment	1 (3.12)	1 (6.25)	1.000	12 (11.76)	7 (13.73)	0.729	21 (12.21)	12 (13.95)	0.693
Aphasia	0 (0.00)	0 (0.00)	NA	3 (2.94)	2 (3.92)	1.000	10 (5.81)	5 (5.81)	1.000
Disturbance of consciousness	0 (0.00)	1 (6.25)	0.721	10 (9.80)	5 (9.80)	1.000	9 (5.23)	5 (5.81)	1.000
**Unilateral/bilateral hematoma**									
Left	15 (46.88)	7 (43.75)	0.966	50 (49.02)	25 (49.02)	0.985	75 (43.60)	38 (44.19)	0.993
Right	10 (31.25)	5 (31.25)		37 (36.27)	18 (35.29)		60 (34.88)	30 (34.88)	
Bilateral	7 (21.88)	4 (25.00)		15 (14.71)	8 (15.69)		37 (21.51)	18 (20.93)	
**Hematoma density**									
Homogeneous	23 (71.88)	12 (75.00)	1.000	57 (55.88)	29 (56.86)	0.908	110 (63.95)	54 (62.79)	0.855
Heterogeneous	9 (28.12)	4 (25.00)		45 (44.12)	22 (43.14)		62 (36.05)	32 (37.21)	
Hematoma volume, ml	110.433 ± 15.817	114.24 ± 17.32	0.450	113.46 (99.24–128.50)	112.34 (101.93–132.00)	0.868	108.07 (94.59–122.11)	108.03 (96.05–123.19)	0.904
**Laboratory investigation**									
Total cholesterol (mmol/L)	3.71 (3.26–4.11)	3.35 (3.08–3.89)	0.213	3.81 (3.30–4.72)	3.72 (3.21–4.32)		3.96 (3.45–4.60)	3.26 (2.90–3.57)	**<0.001**
Platelet (×10^9^/L)	216 (170–244)	114 (101–147)	**<0.001**	215 (187–249)	131 (112–160)	**<0.001**	226 (196–265)	146 (122–185)	**<0.001**
Platelet (×10^9^/L) <125	1 (3.12)	10 (62.50)	**<0.001**	2 (1.96)	22 (43.14)	**<0.001**	1 (0.58)	24 (27.91)	**<0.001**
PT–INR	1.06 (0.98–1.16)	1.09 (1.00–1.21)	0.381	1.04 (0.99–1.11)	1.05 (1.00–1.12)	0.662	1.02 (0.97–1.09)	1.05 (0.99–1.10)	0.153
PT–INR >1.15	11 (34.38)	6 (37.50)	0.831	16 (15.69)	9 (17.65)	0.757	16 (9.30)	10 (11.63)	0.559
APTT (s)	35.8 (32.8–38.5)	36.4 (34.6–38.7)	0.533	37.6 (34.5–40.3)	36.8 (34.1–39.8)	0.558	35.8 (32.9–38.8)	36.0 (33.2–39.0)	0.694
APTT >43 s	1 (3.12)	1 (6.25)	1.000	13 (12.75)	5 (9.80)	0.595	10 (5.81)	5 (5.81)	1.000
AST (IU/L)	20 (17–25)	58 (48–63)	**<0.001**	20 (16–24)	32 (21–49)	**<0.001**	20 (17–25)	21 (17–31)	0.079
AST > 40 IU/L	1 (3.12)	14 (87.50)	**<0.001**	4 (3.92)	19 (37.25)	**<0.001**	8 (4.65)	15 (17.44)	**0.001**
ALT (IU/L)	15 (11–23)	28 (21–44)	**0.001**	14 (10–19)	17 (11–23)	0.275	16 (11–23)	16 (11–21)	0.872
ALT >40 IU/L	3 (9.38)	4 (25.00)	0.311	7 (6.86)	3 (5.88)	1.000	9 (5.23)	4 (4.65)	1.000
GGT (IU/L)	17 (12–38)	15 (9–23)	0.335	14 (10–33)	14 (9–21)	0.338	13 (9–28)	18 (14–41)	**<0.001**
**Recurrence at follow-up**									
1 month	0 (0.00)	2 (12.50)	0.202	4 (3.92)	2 (3.92)	1.000	6 (3.49)	4 (4.65)	0.909
3 months	1 (3.12)	4 (25.00)	0.066	15 (14.71)	9 (17.65)	0.637	19 (11.05)	15 (17.44)	0.152
12 months	3 (9.38)	6 (37.50)	**0.050**	18 (17.65)	18 (35.29)	**0.015**	25 (14.53)	23 (26.74)	**0.018**
Death (in 12 months)	3 (9.38)	1 (6.25)	1.000	2 (1.96)	4 (7.84)	0.077	2 (1.16)	4 (4.65)	0.189

*Bold values indicates statistical significance*.

### Time to Recurrence

**Figure 2:**
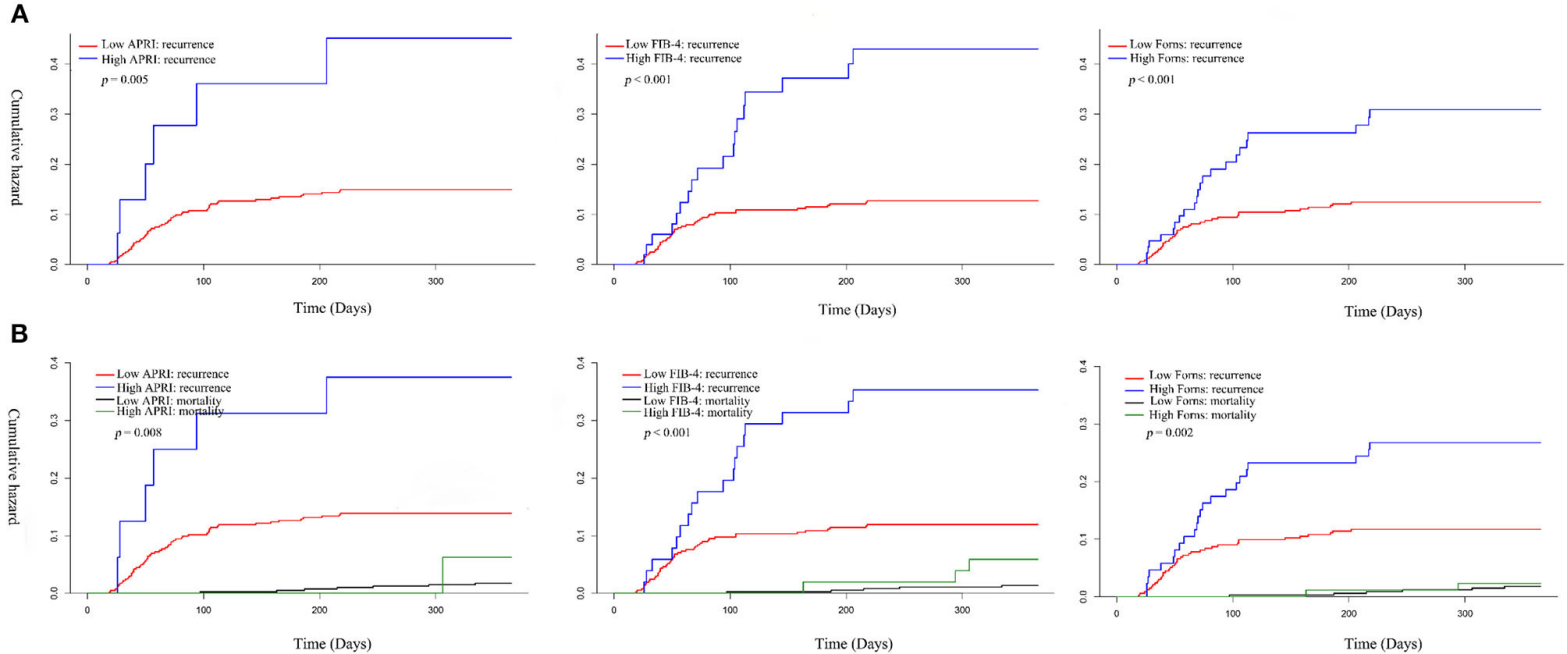
Cumulative hazard of recurrence and competing mortality in patients with CSDH according to low and high Q19 liver fibrosis scores. **(A)** Kaplan–Meier method; **(B)** Kaplan–Meier method.

The overall median time to recurrence was 56 (IQR: 38–103) days, and 72.58 % of the recurrences occurred within 3 months after surgery. There was no significant difference in the recurrence of CSDH either within 1 month or 3 months between the low and high LF scores groups. However, significantly more patients in the high LF scores group experienced recurrence of CSDH within 12 months of surgery. Figure 2A shows the overall cumulative hazard of the recurrence within 12 months from the initial operation for CSDH in the low and the high LF scores groups. Mortality did not seem to have an influence on recurrence rate, because the results were similar when death was used as a competing event (Figure 2B). After adjusting for the confounders, the high level of LF scores remained significantly and independently associated with CSDH recurrence. When included as continuous variables, the LF scores were also associated with an increased risk of CSDH recurrence, with the exception of FIB-4 score (Table 3).

**Table 3:** Associations between liver fibrosis scores and recurrence in patients with CSDH.

**Characteristics**	**Cox regression model**		**Fine–Gray model**		**Logistic regression model**	
	**Adjusteda**	***p***	**Adjusteda**	***p***	**Adjusteda**	***p***
APRI >1.0	4.32 (1.37–13.6)	**0.013**	4.30 (1.07–17.33)	**0.040**	5.30 (1.31–21.41)	**0.019**
FIB-4 >3.25	2.56 (1.20–5.43)	**0.015**	2.56 (1.18–5.54)	**0.017**	3.63 (1.43–9.21)	**0.007**
Forns >6.9	2.02 (1.07–3.79)	**0.029**	2.02 (1.07–3.81)	**0.030**	2.58 (1.21–5.51)	**0.014**
APRI1	4.04 (1.28–12.79)	**0.018**	4.03 (1.04–15.59)	**0.043**	6.68 (1.67–26.71)	**0.007**
FIB-41	1.23 (1.01–1.50)	**0.042**	1.23 (0.99–1.53)	0.065	1.33 (1.04–1.71)	**0.023**
Forns1	1.29 (1.02–1.64)	**0.036**	1.29 (1.01–1.66)	**0.044**	1.38 (1.04–1.82)	**0.024**

*^a^Adjusted for the confounders including age, sex, smoking, drinking, comorbidities, history of head trauma, antiplatelet/anticoagulant therapy, symptoms, laboratory investigation, and CT scan hematoma characteristics*.

*^1^Per unit change in regressor*.

*Bold values indicates statistical significance*.

### Association Between Liver Fibrosis Scores and CSDH Recurrence

**Figure 3:**
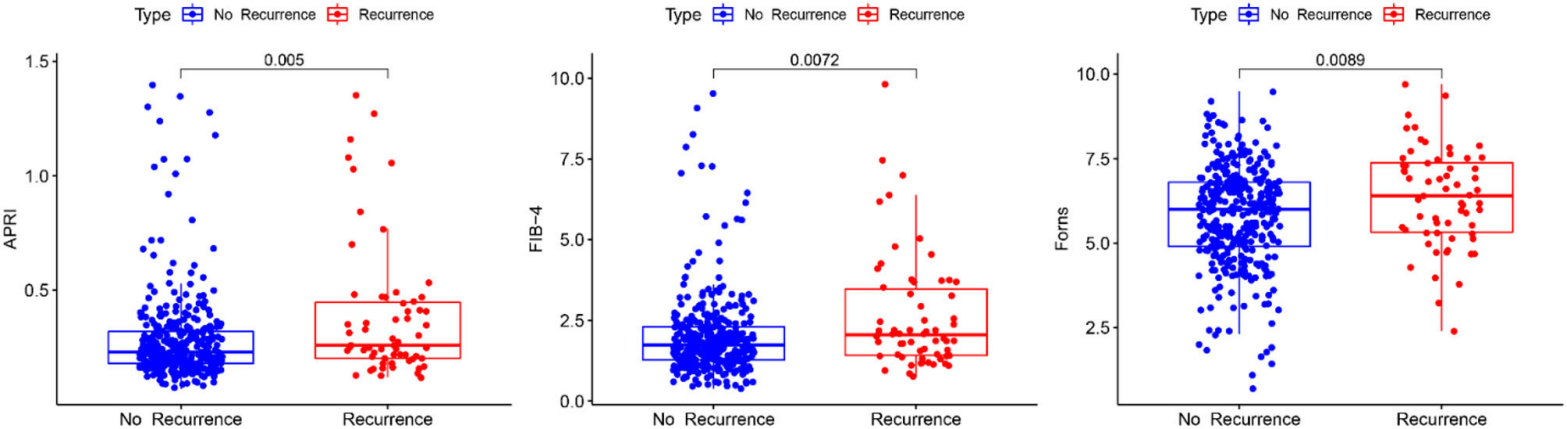
Boxplot of liver fibrosis scores and recurrence in patients with CSDH.

Boxplot analysis revealed a significantly higher APRI, FIB-4, and Forns index scores in the recurrence group than in the no recurrence group (Figure 3). Subsequently, multivariate logistic regression analysis revealed that the LF scores were independent risk factor for the recurrence of CSDH as well (Table 3). We also performed sensitivity analyses excluding patients with drinking history in view of its possible influence. Notably, higher LF scores remained associated with the recurrence of CSDH, consistent with the results of the primary analysis (Supplementary Table S2). To improve the clinical interpretation of the results and allow for a non-linear association between the LF scores and CSDH recurrence, we *a priori* categorized LF scores based on quartiles. According to the trend test, the high LF scores had higher rates of the CSDH recurrence (*p* for trend < 0.05, Table 4). Simultaneously, the dose–response relationship between the LF scores and CSDH recurrence was further demonstrated with RCS method (*p* for non-linearity is more than 0.05, Supplementary Figure S2).

**Table 4:** Trends in the prevalence of CSDH recurrence of the quartiles of liver fibrosis scores.

**Characteristics**	**APRI**				**FIB-4**				**Forns**			
	**Quartile 1**	**Quartile 2**	**Quartile 3**	**Quartile 4**	**Quartile 1**	**Quartile 2**	**Quartile 3**	**Quartile 4**	**Quartile 1**	**Quartile 2**	**Quartile 3**	**Quartile 4**
Recurrence (*n* = 62)	10 (10.42)	12 (10.91)	15 (13.89)	25 (23.81)	10 (9.62)	13 (12.50)	17 (16.04)	22 (20.95)	10 (9.90)	14 (14.74)	11 (10.48)	27 (22.88)
Non-recurrence (*n* = 357)	86 (89.58)	98 (89.09)	93 (86.11)	80 (76.19)	94 (90.38)	91 (87.50)	89 (83.96)	83 (79.05)	91 (90.10)	81 (85.26)	94 (89.52)	91 (77.12)
*p* for trend	**0.016**				**0.034**				**0.038**			

*Bold values indicates statistical significance*.

### Incremental Predictive Value of Liver Fibrosis Scores for CSDH Recurrence

**Figure 4:**
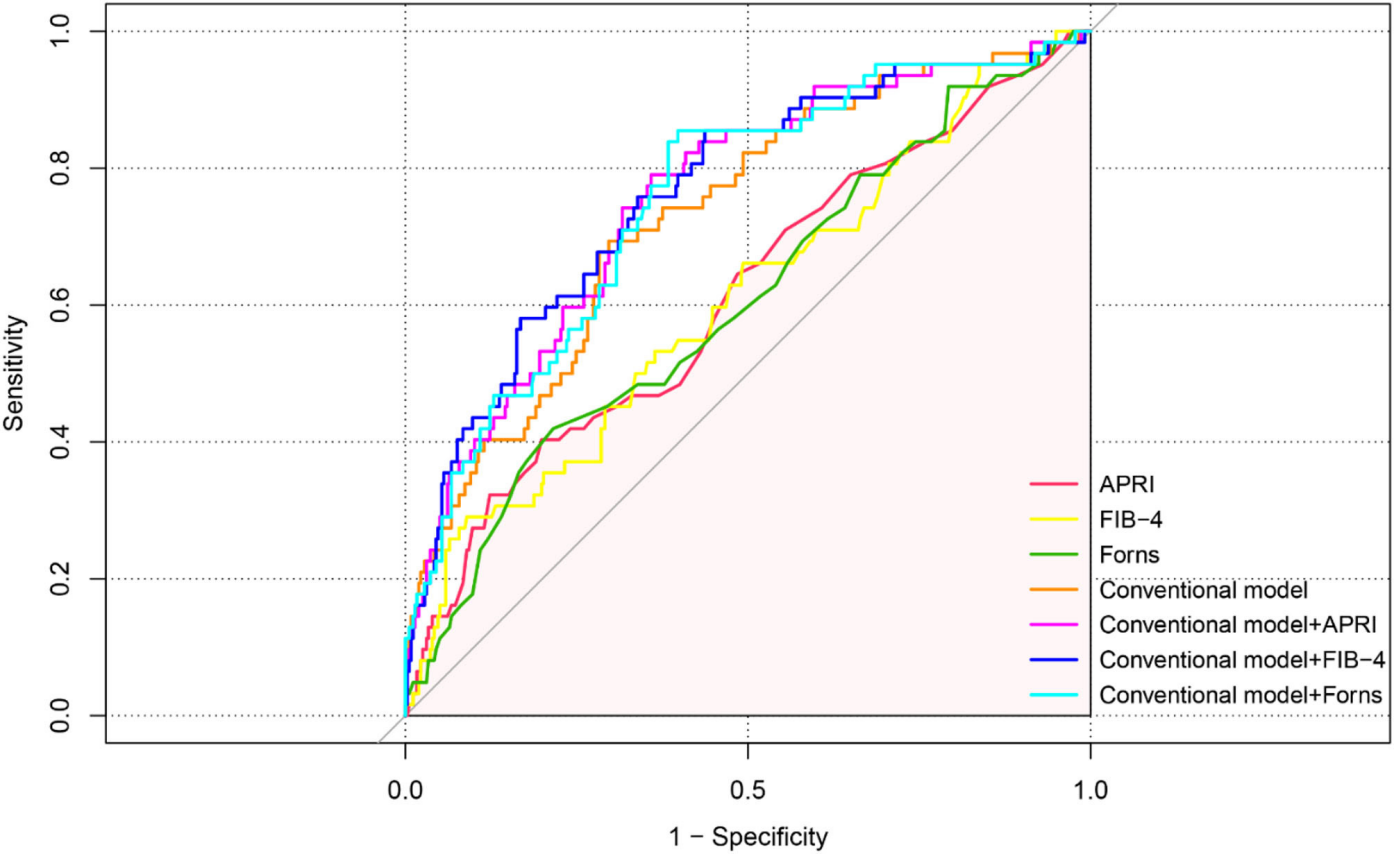
ROC curve analysis of liver fibrosis scores for predicting recurrence in patients with CSDH.

In the ROC analysis shown in Figure 4, APRI, FIB-4, and Forns index scores evaluated separately showed poor–moderate discriminative powers for the recurrence of CSDH (AUC: 0.612, 95% CI: 0.533-0.691, *p* = 0.005 for APRI, AUC: 0.607, 95% CI: 0.528–0.686, *p* = 0.007 for FIB-4 and AUC: 0.604, 95% CI: 0.526–0.682, *p* = 0.009 for Forns index). To further explore the incremental predictive value of the LF scores for CSDH recurrence, we evaluated the effect of adding them to multiparameter CM, respectively. As shown in Table 5, the addition of APRI into the CM allowed a significant incremental prediction of risk for CSDH recurrence (*p* < 0.05, when comparing the two AUC). On the other hand, when the APRI was incorporated into the CM alone, risk reclassification was significantly improved [IDI: 0.0135, 95% CI: −0.0036–0.0307, *p* = 0.121; NRI (Categorical): 0.075, 95% CI: 0.0202–0.1703, *p* = 0.122; NRI (Continuous): 0.5603, 95% CI: 0.2961–0.8245, *p* < 0.001]. Comparable results could be observed by adding another two LF scores to CM for the recurrence of CSDH (Table 5).

**Table 5:** Incremental predictive value of liver fibrosis scores for predicting recurrence in patients with CSDH.

**Characteristics**	**ROC**		**IDI**		**NRI (Categorical)**		**NRI (Continuous)**	
	**AUC (95% CI)**	***p***	**Value (95% CI)**	***p***	**Value (95% CI)**	***p***	**Value (95% CI)**	***p***
Conventional model	0.731 (0.663–0.799)		Refence		Refence		Refence	
Conventional model + APRI	0.763 (0.697–0.830)	**0.037**	0.0135 (−0.0036–0.0307)	0.121	0.075 (−0.0202–0.1703)	0.122	0.5603 (0.2961–0.8245)	**<0.001**
Conventional model + FIB-4	0.755 (0.689–0.822)	**0.048**	0.0194 (−0.0021–0.0409)	0.077	0.1234 (−0.0025–0.2494)	0.054	0.2854 (0.0248–0.5461)	**0.031**
Conventional model + Forns	0.753 (0.687–0.819)	0.155	0.0119 (−0.0037–0.0276)	0.134	0.0407 (−0.0532–0.1345)	0.395	0.3316 (0.0743–0.5889)	**0.011**

*ROC, receiver operating characteristic; NRI, net reclassification improvement; IDI, integrated discrimination improvement*.

*Bold values indicates statistical significance*.

## Discussion

This is the first study to evaluate the association of the LF scores with the recurrence of CSDH after surgery. Our study results showed that higher baseline LF scores, including APRI, FIB-4, and Forns index scores were significantly associated with the postoperative recurrence of CSDH. Moreover, after adjusting for potential confounding variables, high levels of the preceding three LF scores could be an available risk factor for CSDH recurrence. In addition, adding APRI, FIB-4, and Forns index scores to the model of established risk factors significantly improved the risk prediction for CSDH recurrence.

Despite this, there is a growing evidence that the chronic liver disease represents an independent predictor for the poor prognosis of patients with cardio–cerebrovascular diseases, such as coronary atherosclerosis (17, 25), ischemic and hemorrhagic stroke (18, 19, 24), aneurysmal subarachnoid hemorrhage, and also CSDH (11–13, 37). In clinical practices, however, most patients might suffer from subclinical liver disease, in the absence of obvious clinical manifestations or laboratory derangements; for whom regarding the risk of CSDH recurrence are still scarce. Therefore, we hypothesized that subclinical liver disease, defined using the LF scores, is a potentially unrecognized contributor to the recurrence of CSDH after surgery. To test this issue, this study aimed to investigate the association between the LF scores and CSDH recurrence to provide a new orientation to study the pathophysiology of CSDH.

As expected, we found that a significantly higher values for APRI, FIB-4, and Forns index in patients with CSDH recurrence than those without recurrence (all *p* < 0.05). After adjusting for confounding factors by multivariable regression model analysis revealed that higher LF scores was an independent risk factor for CSDH recurrence. The precise mechanisms underlying the association between the LF scores and CSDH recurrence remained unclear. The possible mechanisms may include subclinical coagulopathy, endothelial dysfunction, and vascular inflammation (38–41). First, the liver synthesizes numerous clotting factors involved in the coagulation cascade. Second, thrombopoietin, which dominated the genesis and maturity of platelet, was mostly produced in liver. There is no doubt that coagulation and platelet dysfunction are associated with increased risks of developing chronic hematomas, leading to the postoperative recurrence of CSDH (5, 42). In addition, many studies showed the development of fibrosis is associated with increased inflammatory state, endothelial dysfunction, insulin resistance and lipid metabolism, thereby forming a vicious circle which affects systemic vascular autoregulation and blood vessel walls (17, 21, 25). It is already conceived that a torn bridging vein and neo-membrane bleeding play a pivotal pathological factor for the development and recurrence of CSDH (5, 43). Besides, an increased susceptibility to malnutrition and liver-related complications, as shown in other studies, may provide alternative reasons for the postoperative recurrence of CSDH (19, 44, 45). So, we hypothesized that the presence of advanced fibrosis, even without clinical cirrhosis, may have predisposed to more severe malnutrition or had lesser clinical functional reserve to maintain homeostasis in the follow-up period. However, these mechanisms were mainly described in patients with clinically diagnosed liver disease; whether these mechanisms can be implicated to explain our findings is a hypothesis that requires testing in the future studies. Notably, hematoma recurrence occurred either within 1 or 3 months after surgery was comparable in the high and low LF scores groups, as described in our results. Perhaps, although subclinical liver disease shares some common pathophysiologic mechanisms as confirmed liver diseases, and hematoma recurrence occurs requiring a long incubation period.

Importantly, using LF scores, the composite risk scores including both traditional risk factors and liver-related parameters, had more favorable discrimination ability in predicting the recurrence of CSDH. Such assessments have been developed by combining the various serological parameters that can be easily obtained in routine clinical practice. In other words, understanding that the presence and severity of advanced fibrosis evaluated by non-invasive markers would independently contribute to the risk of CSDH recurrence might raise the awareness for providing timely additional interventions to liver health and improve the prognosis in patients with CSDH.

However, several limitations should be noted for this study. First, data from the APRI, FIB4, and Forns index scores were available only at baseline. There was no information regarding fibrosis changes during follow-up. Generally, longitudinal trajectories and dynamic change of the LF scores could better select individuals at risk for the recurrence of CSDH. Second, although we purposefully excluded the patients who consumed excessive alcohol and diagnosed liver diseases, we cannot completely rule out the existence of unrecognized liver diseases in the study participants because liver biopsies were not taken in the evaluation of fibrosis. Third, although multiple regression analysis and PSM analysis were used to minimize the confounding effects from those known parameters, these LF scores used in this study include relatively non-specific metrics, such as age, platelet count and total cholesterol. Hence, the extent to which LF itself is responsible for our findings, as opposed to being an epiphenomenon related to the established risk factors (3, 5, 46), requires further investigation. In addition, we were not able to investigate all other LF scores indices. However, given the robust results of preceding three LF scores, it seems unlikely that the analysis of additional scores would have yielded diverging results. Finally, this study was only a retrospective single-center study and as such, it might have several bias and variation. Hence, further prospective, multicenter studies are still needed to validate our findings in this study.

## Conclusions

In conclusion, our study demonstrated that the higher LF scores (APRI, FIB-4, and Forns index score), as a simple and invasive assessment, were significantly associated with the postoperative recurrence in patients with CSDH. These findings may provide novel perspectives in cerebrovascular-liver clinical practice and support the notion that the LF scores might be novel tools to identify patients at high risk for the recurrence of CSDH. Certainly, further studies are needed to confirm these findings and to investigate the mechanisms between subclinical liver disease and CSDH recurrence.

## Data Availability Statement

The raw data that support the findings of this study are available from the First Affiliated Hospital of Wenzhou Medical University, but restrictions apply with regard to the availability of the data which were used under license for the current study and are not publicly available. Data are however available from the authors upon reasonable request and with permission of the First Affiliated Hospital of Wenzhou Medical University.

## Author Contributions

LR, QZ, and PZ contributed to conception and design of the study. PZ, HW, and HB wrote the first draft of the manuscript. NW, ZC, and QT wrote sections of the manuscript. XL, YL, ZZ, and YC organized the data collection. PZ, HW, QT, and NW performed the statistical analysis. All authors contributed to manuscript revision, read, and approved the submitted version.

## Funding

This study was funded by the General Scientific Research Projects of Zhejiang Provincial Department of Education (Grant No. Y202147811).

## Conflict of Interest

The authors declare that the research was conducted in the absence of any commercial or financial relationships that could be construed as a potential conflict of interest.

## Publisher's Note

All claims expressed in this article are solely those of the authors and do not necessarily represent those of their affiliated organizations, or those of the publisher, the editors and the reviewers. Any product that may be evaluated in this article, or claim that may be made by its manufacturer, is not guaranteed or endorsed by the publisher.
