# Supplementary material for: Non-invasive Liver Fibrosis Scores Are Associated With Recurrence of Postoperative Chronic Subdural Hematoma
Source: Front Neurol. 2022 Jun 13;13:873124. doi: 10.3389/fneur.2022.873124 (PMC9236226; doi:10.3389/fneur.2022.873124)
Supplement: Supplementary file 1 [file Data_Sheet_1.docx]

**Supplementary Table 1.** Baseline characteristics and outcomes of patients with CSDH, stratified by liver fibrosis scores before propensity score matching

| Characteristics | Low APRI | High APRI | *p* value | Low FIB-4 | High FIB-4 | *p* value | Low Forns | High Forns | *p* value |
| --- | --- | --- | --- | --- | --- | --- | --- | --- | --- |
|  | n = 403 | n = 16 |  | n = 368 | n = 51 |  | n = 333 | n = 86 |  |
| Age | 72 (64-80) | 76 (73-81) | 0.184 | 71 (63-79) | 80 (75-85) | **<0.001** | 71 (63-79) | 77 (71-83) | **<0.001** |
| Gender |  |  |  |  |  |  |  |  |  |
| Female | 62 (15.38) | 2 (12.50) | 1.000 | 56 (15.22) | 8 (15.69) | 0.930 | 54 (16.22) | 10 (11.63) | 0.292 |
| Male | 341 (84.62) | 14 (87.50) |  | 312 (84.78) | 43 (84.31) |  | 279 (83.78) | 76 (88.37) |  |
| Personal/Past history |  |  |  |  |  |  |  |  |  |
| Smoking | 162 (40.20) | 6 (37.50) | 0.829 | 148 (40.22) | 20 (39.22) | 0.891 | 128 (38.44) | 40 (46.51) | 0.173 |
| Drinking | 97 (24.07) | 3 (18.75) | 0.849 | 92 (25.00) | 8 (15.69) | 0.144 | 73 (21.92) | 27 (31.40) | 0.066 |
| Hypertension | 162 (40.20) | 7 (43.75) | 0.776 | 143 (38.86) | 26 (50.98) | 0.098 | 133 (39.94) | 36 (41.86) | 0.746 |
| Diabetes | 50 (12.41) | 2 (12.50) | 1.000 | 44 (11.96) | 8 (15.69) | 0.449 | 38 (11.41) | 14 (16.28) | 0.222 |
| Cardiac diseases | 30 (7.44) | 1 (6.25) | 1.000 | 22 (5.98) | 9 (17.65) | **0.007** | 17 (5.11) | 14 (16.28) | **<0.001** |
| Head injury | 276 (68.49) | 11 (68.75) | 0.982 | 259 (70.38) | 28 (54.90) | **0.026** | 235 (70.57) | 52 (60.47) | 0.072 |
| Antiplatelet/anticoagulant therapy | 77 (19.11) | 4 (25.00) | 0.793 | 67 (18.21) | 14 (27.45) | 0.117 | 63 (18.92) | 18 (20.93) | 0.674 |
| The main symptoms |  |  |  |  |  |  |  |  |  |
| Headache and/or dizziness | 225 (55.83) | 10 (62.50) | 0.598 | 207 (56.25) | 28 (54.90) | 0.856 | 182 (54.65) | 53 (61.63) | 0.245 |
| Limb weakness | 181 (44.91) | 9 (56.25) | 0.372 | 169 (45.92) | 21 (41.18) | 0.523 | 156 (46.85) | 34 (39.53) | 0.225 |
| Disorientation/memory impairment | 43 (10.67) | 1 (6.25) | 0.881 | 37 (10.05) | 7 (13.73) | 0.423 | 32 (9.61) | 12 (13.95) | 0.241 |
| Aphasia | 19 (4.71) | 0 (0.00) | 0.782 | 17 (4.62) | 2 (3.92) | 1.000 | 14 (4.20) | 5 (5.81) | 0.727 |
| Disturbance of consciousness | 32 (7.94) | 1 (6.25) | 1.000 | 28 (7.61) | 5 (9.80) | 0.789 | 28 (8.41) | 5 (5.81) | 0.426 |
| Unilateral/bilateral hematoma |  |  |  |  |  |  |  |  |  |
| Left | 185 (45.91) | 7 (43.75) | 0.890 | 167 (45.38) | 25 (49.02) | 0.681 | 154 (46.25) | 38 (44.19) | 0.943 |
| Right | 137 (34.00) | 5 (31.25) |  | 124 (33.70) | 18 (35.29) |  | 112 (33.63) | 30 (34.88) |  |
| Bilateral | 81 (20.10) | 4 (25.00) |  | 77 (20.92) | 8 (15.69) |  | 67 (20.12) | 18 (20.93) |  |
| Hematoma density |  |  |  |  |  |  |  |  |  |
| Homogeneous | 280 (69.48) | 12 (75.00) | 0.846 | 263 (71.47) | 29 (56.86) | **0.033** | 238 (71.47) | 54 (62.79) | 0.118 |
| Heterogeneous | 123 (30.52) | 4 (25.00) |  | 105 (28.53) | 22 (43.14) |  | 95 (28.53) | 32 (37.21) |  |
| Hematoma volume, mL | 108.32 (94.21-124.52) | 115.84 (100.19-125.05) | 0.352 | 108.13 (93.61-123.41) | 112.34 (101.93-132.00) | 0.103 | 109.20 (93.80-125.17) | 108.03 (96.05-123.19) | 0.615 |
| Laboratory investigation |  |  |  |  |  |  |  |  |  |
| Total cholesterol (mmol/L) | 3.76 (3.26-4.44) | 3.35 (3.08-3.89) | 0.115 | 3.76 (3.25-4.46) | 3.72 (3.21-4.32) | 0.593 | 3.84 (3.39-4.54) | 3.26 (2.90-3.57) | **<0.001** |
| Platelet (×10^9/L) | 217 (178-253) | 114 (101-147) | **<0.001** | 223 (188-257) | 131 (112-160) | **<0.001** | 228 (193-264) | 146 (122-185) | **<0.001** |
| Platelet (×10^9/L) < 125 | 16 (3.97) | 10 (62.50) | **<0.001** | 4 (1.09) | 22 (43.14) | **<0.001** | 2 (0.60) | 24 (27.91) | **<0.001** |
| PT-INR | 1.02 (0.96-1.07) | 1.09 (1.00-1.21) | **0.012** | 1.02 (0.96-1.07) | 1.05 (1.00-1.12) | **0.009** | 1.01(0.96-1.07) | 1.05 (0.99-1.10) | **0.002** |
| PT-INR > 1.15 | 24 (5.96) | 6 (37.50) | **<0.001** | 21 (5.71) | 9 (17.65) | **0.005** | 20 (6.01) | 10 (11.63) | 0.071 |
| APTT (s) | 36.1 (33.3-39.3) | 36.4 (34.6-38.7) | 0.925 | 36 (33.3-39.1) | 36.8 (34.1-39.8) | 0.267 | 36.2 (33.4-39.4) | 36.0 (33.2-39.0) | 0.439 |
| APTT > 43 s | 32 (7.94) | 1 (6.25) | 1.000 | 28 (7.61) | 5 (9.80) | 0.789 | 28 (8.41) | 5 (5.81) | 0.426 |
| AST (IU/L) | 20 (17-24) | 58 (48-63) | **<0.001** | 20 (17-24) | 32 (21-49) | **<0.001** | 20 (17-24) | 21 (17-31) | **0.011** |
| AST > 40 IU/L | 12 (2.98) | 14 (87.50) | **<0.001** | 7 (1.90) | 19 (37.25) | **<0.001** | 11 (3.30) | 15 (17.44) | **<0.001** |
| ALT (IU/L) | 15 (10-21) | 28 (21-44) | **<0.001** | 15 [11-21) | 17 (11-23) | 0.320 | 15 (10-21) | 16 (11-21) | 0.419 |
| ALT > 40 IU/L | 14 (3.47) | 4 (25.00) | **<0.001** | 15 (4.08) | 3 (5.88) | 0.820 | 14 (4.20) | 4 (4.65) | 1.000 |
| GGT (IU/L) | 14 (10-30) | 15 (9-23) | 0.968 | 14 (10-31) | 14 (9-21) | 0.338 | 13 (9-26) | 18 (14-41) | **<0.001** |
| Recurrence at follow-up |  |  |  |  |  |  |  |  |  |
| 1 month | 8 (1.99) | 2 (12.50) | 0.062 | 8 (2.17) | 2 (3.92) | 0.782 | 6 (1.80) | 4 (4.65) | 0.251 |
| 3 months | 41 (10.17) | 4 (25.00) | 0.142 | 36 (9.78) | 9 (17.65) | 0.089 | 30 (9.01) | 15 (17.44) | **0.024** |
| 12 months | 56 (13.90) | 6 (37.50) | **0.025** | 44 (11.96) | 18 (35.29) | **<0.001** | 39 (11.71) | 23 (26.74) | **<0.001** |
| Death (in 12 months) | 10 (2.48) | 1 (6.25) | 0.899 | 7 (1.90) | 4 (7.84) | **0.043** | 7 (2.10) | 4 (4.65) | 0.347 |

Boldface type indicates statistical significance.

**Supplementary Table 2.** Associations between liver fibrosis scores and CSDH recurrence after excluding patients with drinking history

| Characteristics | Cox regression model | | Fine and Gray model | | Logistic regression model | |
| --- | --- | --- | --- | --- | --- | --- |
|  | Adjusted^a^ | *p* value | Adjusted^a^ | *p* value | Adjusted^a^ | *p* value |
| APRI > 1.0 | 7.58 (1.94-29.61) | **0.004** | 5.49 (1.73-17.44) | **0.004** | 15.90 (2.91-86.97) | **0.001** |
| FIB-4 > 3.25 | 3.09 (1.30-7.36) | **0.011** | 2.64 (1.33-5.24) | **0.006** | 4.50 (1.47-13.76) | **0.008** |
| Forns > 6.9 | 2.05 (0.97-4.35) | 0.061 | 2.13 (1.17-3.86) | **0.013** | 2.61 (1.04-6.60) | **0.042** |
| APRI^1^ | 6.30 (1.62-24.54) | **0.008** | 5.50 (1.72-17.53) | **0.004** | 13.74 (2.58-73.06) | **0.002** |
| FIB-4^1^ | 1.28 (1.03-1.60) | **0.027** | 1.28 (1.05-1.55) | **0.014** | 1.45 (1.08-1.94) | **0.012** |
| Forns^1^ | 1.39 (1.03-1.87) | **0.029** | 1.42 (1.12-1.79) | **0.003** | 1.57 (1.09-2.25) | **0.014** |

^a^Adjusted for the confounders including age, sex, smoking, drinking, comorbidities, history of head trauma, antiplatelet/anticoagulant therapy, symptoms, laboratory investigation, and CT scan hematoma characteristics.

^1^Per unit change in regressor.

Boldface type indicates statistical significance.


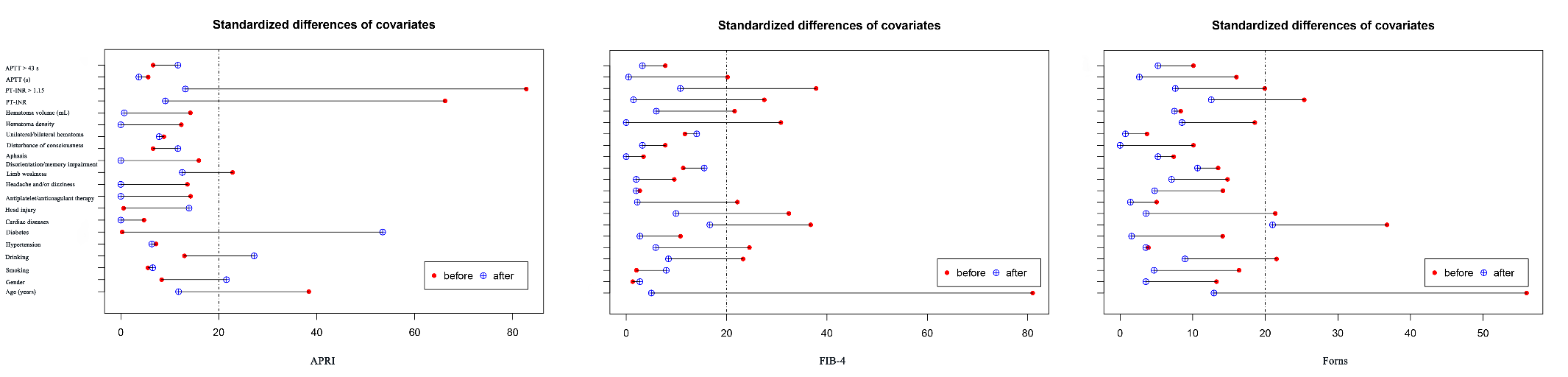


**Supplementary Figure 1.** Absolute standardized differences before and after propensity score matching comparing variables values between patients with high and low liver fibrosis scores.


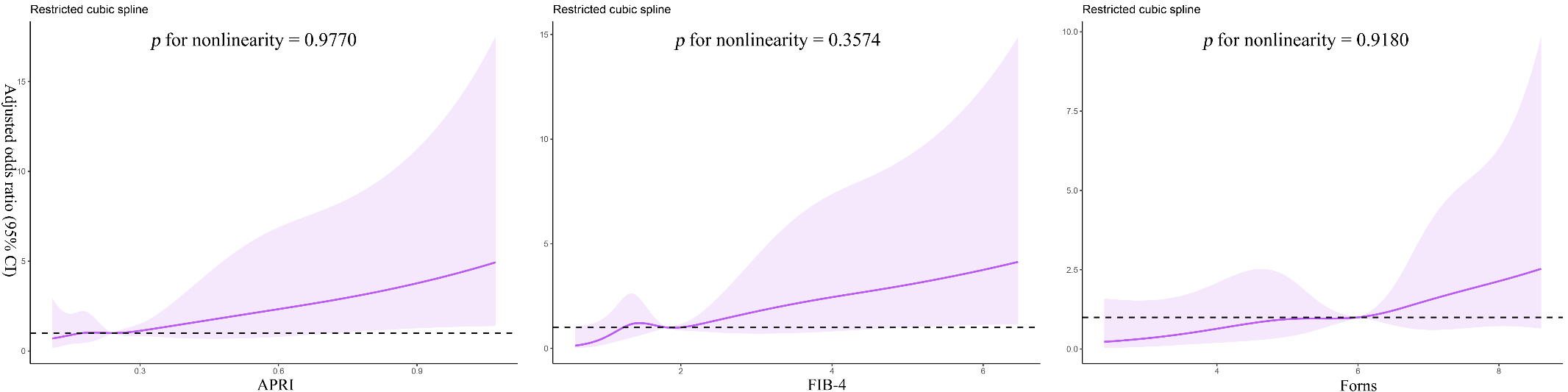


**Supplementary Figure 2.** Restricted cubic spline of the relationship between liver fibrosis scores and recurrence in patients with CSDH.
